# Is the Presence of eCAP Intraoperatively a Predictor of Cochlear Implant Outcomes in Adults?

**DOI:** 10.1055/s-0046-1820088

**Published:** 2026-09-11

**Authors:** Georgea Espindola Ribeiro, Maria Valéria Schmidt Goffi-Gomez, Ana Tereza de Matos Magalhães, Ana Cristina Hiromi Hoshino, Robinson Koji Tsuji

**Affiliations:** 1 Teaching Hospital, Faculdade de Medicina de Botucatu, Universidade Estadual Paulista “Júlio de Mesquita Filho” (UNESP), São Paulo, SP, Brazil; 2 Central Institute, Hospital das Clínicas, Faculdade de Medicina, Universidade de São Paulo (ICHC FMUSP), São Paulo, SP, Brazil; 3 Instituto de Estudos em Saúde Coletiva, Universidade Federal do Rio de Janeiro, Rio de Janeiro, RJ, Brazil

**Keywords:** cochlear implant, electrical compound action potential, electrical stimulation, objective measures, speech recognition, neural response

## Abstract

**Introduction:**

The electrically-evoked compound action potential (eCAP) is commonly used to assess the physiological integrity of the auditory nerve's first neuron in cochlear implant (CI) users. Electrical stimulation of this neuron is essential for transmitting auditory signals to the auditory cortex, enabling the recognition of acoustic cues.

**Objective:**

To determine whether the presence of intraoperative eCAP influences CI outcomes in adult individuals with pre- or postlingual deafness.

**Methods:**

The present retrospective cross-sectional study included adults with hearing loss of various etiologies who received a CI, either with pre- or postlingual deafness. Data on intraoperative eCAP results and speech recognition test outcomes after 12 months of consistent CI use were extracted from medical records.

**Results:**

Data from 121 ears were analyzed, comprising 101 individuals with post- and 20 with prelingual deafness. In the overall sample, 90.9% exhibited eCAP presence intraoperatively. In the prelingual group, eCAP was present in 100% of cases, while 89% of the postlingual group showed eCAP presence. Individuals with intraoperative eCAP demonstrated significantly better speech recognition performance compared with those without eCAP, in both open- and closed-set tests.

**Conclusion:**

In adults with postlingual deafness, 89% exhibited intraoperative eCAP, which correlated with significantly better speech recognition outcomes. In contrast, the prelingual group demonstrated intraoperative neural responses, but no significant correlation with speech recognition performance was observed.

## Introduction

The cochlear implant (CI) is a surgically-implanted device for individuals with severe or profound bilateral sensorineural hearing loss, who receive limited benefit from conventional hearing aids. 1 The device uses electrodes inserted into the cochlea to stimulate the auditory nerve at the spiral ganglion, respecting the cochlear tonotopic organization. 2 3

The stimulation of nerve fibers generates the electrically evoked compound action potential (eCAP), which can be recorded via neural response telemetry (NRT). 4 5 The eCAP has been used to verify the physiological integrity of the auditory nerve immediately after insertion into the cochlea, to assess neural responses across different electrodes along the cochlea, and to predict stimulation levels for mapping. Additionally, it is a quick method that does not depend on patient cooperation, allowing potential recording both intra- and postoperatively. 6 7 8 9 10

Neural response telemetry is the applied method that enables the capture of eCAP. Each brand of CI has its own method for recording and canceling artifacts, with different names for the recorded neural responses. 4 The presence of eCAP has been interpreted as indicative of auditory nerve fiber activation, suggesting a potential for effective transmission of temporal and spectral information. 9 This condition could theoretically contribute to improved performance in auditory skills, such as speech recognition; however, this association is not yet fully established in the literature, and its interpretation requires caution.

Although it is a measure that assesses peripheral auditory structures, it is known that electrical stimulation along this pathway is crucial for the stimulus to reach the auditory cortex. Furthermore, the lack of this information may hinder the mapping process. 7 9 11 12

Another relevant aspect to consider is the investigation of neural response behavior in individuals with prelingual hearing loss who receive a CI in adulthood. The literature suggests that long-term sensory deprivation can reduce the number of ganglion cells, leading to the hypothesis that these individuals may exhibit a higher rate of NRT failure intraoperatively, as well as derive less benefit from the CI. 9 13 14

The present study aimed to determine whether the presence of intraoperative eCAP influences CI outcomes in adults with pre- and postlingual hearing loss.

## Methods

The present is a retrospective cross-sectional study, approved by the institution's Research Ethics Committee, process CAAE: 03409212.8.0000.0068.

Data were collected through a review of medical records of individuals who received a CI between January 2022 and December 2023, provided by the CI group of a tertiary care hospital.

## Sample

### Inclusion Criteria

- Medical records of individuals of both genders with hearing loss of various etiologies who received the CI in adulthood, with pre- or postlingual hearing loss complete insertion of the electrodes into the cochlea, and effective use of the CI (more than 8 hours daily).

### Exclusion Criteria

- Suspected etiology related to auditory neuropathy spectrum disorder or neural desynchrony; individuals who, for any reason, whether cognitive or neurological, could not participate in all the procedures involved.

### Grouping

Participants were divided into two groups according to the presence or absence of intraoperative neural response (eCAP):

- Group 1: presence of eCAP in at least one of the electrodes tested.
- Group 2: absence of eCAP in all electrodes tested.

### Recording eCAP via NRT

As part of the routine procedure for the CI group, NRT testing is performed on all patients during the intraoperative period.

**Fig. 1:**
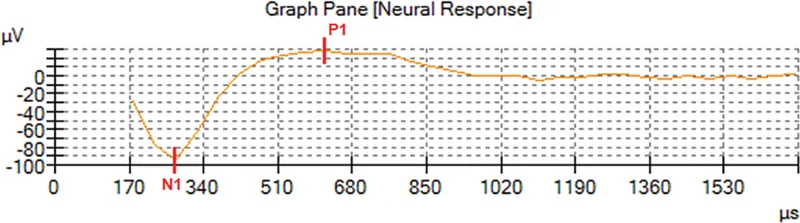
Demonstration of the presence of the Electrically Evoked Compound Action Potential (eCAP).

To record eCAP, a train of electrical pulses is delivered to an intracochlear electrode, and the neural response is recorded by an adjacent electrode. This allows a direct measurement of the auditory nerve's response to electrical stimulation, reflecting the synchronized firing of auditory nerve fibers, similar to the wave I observed in the auditory brainstem response (ABR), occurring at a latency of less than 0.5 milliseconds. 15 Conventionally, the waveform consists of an initial negative peak followed by a positive peak, referred to as N1 and P1, respectively ( Fig. 1 ). These responses are obtained immediately after stimulus presentation, without interference from auditory system maturation. However, the greater the number of neurons recruited in generating the electric field, the larger the amplitude of this potential. 4

The eCAP growth function is recorded at various stimulation current levels, and once the N1 wave is identified (with expected latencies between 0.2–0.4 milliseconds) and the P1 wave (with latencies up to 1 millisecond), the threshold is defined as the lowest stimulus current that produces a neural response. 16 This recording is obtained via software that automatically captures the response, using different techniques depending on the CI manufacturer.

### Classification Criteria and Variables of Interest

The variables selected for analysis were those considered relevant for understanding the association between the presence of intraoperative neural response (eCAP) and speech perception performance with CIs. The following variables were included:

- **Duration of hearing loss:** classified as pre- or postlingual, based on patient report and clinical history.
- **Presence or absence of intraoperative neural response (eCAP):** as previously defined, *absent* was considered when no response was identified in any of the electrodes evaluated, even under maximum current stimulation and after protocol optimization. When at least one electrode exhibited a response, the outcome was considered *present* .
- **Speech perception performance in quiet:** assessed using the Portuguese Sentence Identification Test, 17 recorded as the percentage of correct responses under open- and closed-set presentation conditions.
- **Etiology of hearing loss:** obtained from medical records and categorized according to available data (e.g., genetic, infectious, idiopathic).
- **Duration of auditory deprivation prior to implantation:** defined as the period (in years) between the reported onset of profound hearing loss and the date of CI surgery.
- **Previous use of hearing aids (HA):** self-reported information indicating whether the patient used HAs continuously from the time of diagnosis until entry into the CI program.
- **Residual hearing prior to implantation:** considered present when detection or discrimination of speech was possible with HAs, even under closed-set conditions.

### Speech Perception Test

Speech perception assessment was conducted after 12 months of continuous CI use (more than 8 hours daily), without lipreading or the use of signs/gestures. The Portuguese Sentence Identification Test 17 was employed, consisting of 10 lists with 10 sentences each, pre-recorded by an adult speaker and presented in free-field at 65 dB SPL, in an acoustically-treated booth. The test was initially administered in an open-set condition, without prior presentation of the sentences. In cases in which participants scored ≤ 20% correct in open-set sentence identification, an additional list was administered in a closed-set condition, in which the sentences were displayed on a screen and the participant was required to select the sentence corresponding to the one they had heard. Performance was recorded as the percentage of correct responses in each condition, ranging from 0 to 100%.

### Statistical Analysis

Data were analyzed using descriptive statistics, and the comparison of speech recognition values between individuals with absence and presence of eCAP was performed using the Mann-Whitney test, a non-parametric test for independent samples. Statistical significance was considered at *p* < 0.05.

## Results

### Demographic and Clinical Characteristics of the Sample

The sample consisted of 121 ears from 121 participants, including 101 with postlingual deafness and 20 with prelingual deafness. Age ranged from 18 to 85 years, with a median of 52 years in the postlingual group and 32 (range: 18–42) years in the prelingual group. The duration of auditory deprivation varied widely, being longer in prelingual cases. Most participants reported previous hearing aid (HA) use, and approximately two thirds demonstrated residual hearing prior to surgery ( Table 1 ).

**Table 1:** Demographic distribution of the sample studied ( *n* = 121 ears)

	Prelingual HL	Postlingual HL	Total
**Number of participants**	20	101	121
**Age (years)** Median (min–max)	32 (18–42)	52 (18–85)	49 (18–85)
**Preimplantation deprivation time (years)** Median (min–max)			
Right ear	32 (15–43)	17 (0–54)	–
Left ear	32 (15–43)	18 (0–70)	–
**Use of hearing aid in preimplantation (n)**			
Right ear	16	62	78
Left ear	17	60	77
**Preimplantation PTA (dB HL)** Median (min- max)			
Right ear	90 (90–95) 105 (65–130)		
Left ear	105 (90–120) 110 (80–130)		
**Preimplantation auditory residue (n)**			
Right ear	12	62	74
Left ear	14	66	80
**Preimplantation speech rehabilitation (n)**	9	22	31
**Cochlear implant (n)**			
Right ear	11	64	
Left ear	9	37	

**Abbreviations:** 130 dBHL, absence of responses at the maximum output of the device at frequencies of 500, 1,000, and 2,000 Hz; HL, hearing loss; max, maximum value; min, minimum value; n, number of participants; PTA, pure tone average.

The etiologies were diverse, with idiopathic causes predominating in the postlingual group and congenital causes in the prelingual group. The most frequent etiologies were of unknown origin. Three cochlear implant brands were used (Cochlear Limited , Advanced Bionics, and MED-EL), with similar distribution across groups ( Table 2 ).

**Table 2:** Demographic distribution of the sample studied by etiology and manufacturer of the cochlear implant

	Prelingual HL	Postlingual HL	Total
**Number of participants**	20	101	121
**Etiology**	**n (%)**	**n (%)**	**Total n (%)**
Idiopathic	3 (15)	42 (41.6)	44 (36,6)
Meningitis	4 (20)	8 (8)	13 (10.7)
Otosclerosis	0 (0)	13 (13)	13 (10.7)
Traumatic brain injury	0 (0)	11(11)	11(9)
Infectious	5 (25)	4 (4)	9 (7.4)
Unknown congenital	8 (40)	0 (0)	8 (6.6)
Hereditary	0 (0)	7 (7)	7 (5.8)
Sudden	0 (0)	7 (7)	7 (5.8)
Ototoxicity	0 (0)	3 (3)	3 (2.4)
Autoimmune	0 (0)	3 (3)	3 (2.4)
Usher syndrome	0 (0)	1 (1)	1 (0.8)
Susac syndrome	0 (0)	1 (1)	1(0.8)
Enlarged vestibular aqueduct	0 (0)	1 (1)	1 (0.8)
**CI Model (N)**	**n (%)**	**n (%)**	**Total n (%)**
Cochlear	11 (55)	49 (48.5)	60 (49.5)
Advanced Bionics	8 (40)	29 (28.7)	37 (30.5)
Medel	1 (5)	23 (22.7)	24 (19.8)

**Abbreviations:** HL, hearing loss; CI, cochlear implant; n, number of participants.

### Presence of Intraoperative eCAP

Intraoperative neural response (eCAP) was recorded in 110 of the 121 cases (90.9%). All participants with prelingual deafness exhibited eCAP (100%), whereas 89% of postlingual participants demonstrated responses. Distribution by CI brand is shown in Table 3 .

**Table 3:** Number of patients with presence and absence of eCAP intraoperatively in the sample studied, distributed by the respective cochlear implant brands

	Prelingual HL	Postlingual HL	Total
	N (%)	N (%)	N (%)
Presence of eCAP total	20 (100)	90 (89.1)	110 (90.9)
Cochlear	11 (18.3)	44 (73.4)	55 (91.7)
Advanced Bionics	8 (21.6)	26 (70.3)	34 (91.9)
Medel	1 (4.2)	20 (83.3)	21 (87.5)
Absence of eCAP total	0	11 (10.9)	11 (9.1)
Cochlear ( *N* = 60)	–	5 (8.3)	5 (8.3)
Advanced Bionics ( *N* = 37)	–	3 (8.1)	3 (8.1)
Medel ( *N* = 24)	–	3 (12.5)	3 (12.5)

**Abbreviations:** eCAP, electrically evoked composite action potential; HL, hearing loss.

### Clinical Characteristics of Cases Without eCAP

Absence of eCAP was observed exclusively in the postlingual group ( *n* = 11). Etiologies were varied, including progressive idiopathic hearing loss, meningitis, infection, trauma, and Usher syndrome. Age ranged from 32 to 85 years (median = 58), and the duration of auditory deprivation ranged from 1 to 70 years. Most individuals reported irregular HA use in the implanted ear, and only one had undergone formal auditory rehabilitation prior to implantation ( Table 4 ).

**Table 4:** Distribution of variables etiology, chronological age, and duration of auditory deprivation in patients with postlingual hearing loss

Subject		Deafness (in years)	Chronological age (in years)
**S1**	TBI	2	59
**S2**	Usher syndrome with hearing loss starting in adolescence	10	63
**S3**	Progressive/Idiopathic	6	44
**S4**	Progressive/Idiopathic	5	66
**S5**	Progressive/Idiopathic	10	61
**S6**	Progressive/Idiopathic	21	32
**S7**	Infectious	70	85
**S8**	Meningitis	1	50
**S9**	Progressive genetic	13	58
**S10**	Progressive/Idiopathic	15	81
**S11**	Infectious	36	47

**Abbreviations:** CI, cochlear implant; S, subject; TBI, traumatic brain injury.

### Speech Recognition Test Performance

Out of the 121 ears, 14 did not contain complete information on speech recognition after 12 months of CI use, for various reasons such as dropout, failure to attend the annual evaluation, or abandonment of use. Therefore, 107 ears were included in the analysis of the correlation between speech recognition and the presence or absence of eCAP.

### Postlingual Deafness

After 12 months of CI use, among the 101 individuals with postlingual deafness, 91 completed the speech recognition tests: 81 with present eCAP and 10 with absent eCAP.

In the closed-set condition, the group with eCAP achieved a median score of 100% correct responses, whereas the group without eCAP achieved a median of 45%, demonstrating a significant difference between groups ( *p* = 0.0046). In the open-set condition, median scores were 70% for the group with eCAP and 10% for the group without eCAP ( *p* = 0.0281), indicating a statistically significant association between the presence of eCAP and better performance ( Table 5 ).

**Table 5:** Median speech recognition (in %) in open-set presentation ( *N* = 107 ears)

	Postlingual		*p*
	eCAP presence	eCAP absence	
**N**	81	10	
**Closed-set presentation** Med (min–max)	100 (0–100)	45 (0–100)	0.0046*
**Open-set presentation** Med (min–max)	70 (0–100)	10 (0–100)	0.0281*
	**Prelingual**		
	eCAP presence	eCAP absence	
**N**	16	0	
**Closed-set presentation** Med (min–max)	55 (0–100)	–	–
**Open-set presentation** Med (min–max)	0 (0–50)	–	–

**Abbreviations:** max, maximum value; med, median; min, minimum value.

**Note:** * *p* indicates statistical significance (Mann-Whitney test).

### Prelingual Deafness

Of the 20 individuals with prelingual deafness, 16 completed the speech perception tests. All exhibited intraoperative eCAP. In the closed-set condition, the median score was 55% correct responses. In the open-set condition, the median was 0%, ranging from 0 to 50%. Only 4 individuals (20%) demonstrated sentence recognition in the open-set condition, with scores ranging from 20 to 50% ( Table 5 ).

No significant correlation was observed between the presence of eCAP and test performance, since all participants exhibited neural responses.

## Discussion

The literature indicates that although the presence of eCAP during the intraoperative period does not guarantee that cortical structures are efficiently activated to provide sound perception with CI use, 7 the presence of the eCAP ensures that electrical information is being efficiently transmitted to the auditory cortex. 11 18

The literature shows that long-term sensory deprivation can reduce the number of ganglion cells. 14 This leads us to consider that this population may present a higher rate of eCAP absence compared with postlingual individuals due to neuronal degeneration. 19 However, in this study, all prelingual participants exhibited eCAP during the intraoperative period, regardless of the etiology of hearing loss, duration of auditory deprivation, or CI manufacturer. In the sample studied, 75% of these participants were effective users of hearing aids, and more than half had some residual hearing. In other words, these patients had some measurable speech-sound perception with hearing aid use, even in closed-set presentation.

Histological exams indicate that the longer the duration of hearing loss, the greater the neural degeneration of spiral ganglion cells. 20 However, other research suggests that this may not necessarily compromise these structures in a radical way. 15 19 Thus, it is suggested that the effective use of hearing aids and some level of access to speech sounds, even in closed-set presentation, allows for the maintenance of afferent neural pathway due to the constant stimulation of the peripheral and central auditory pathways. This may have contributed to the detection of eCAP in all participants within this group. 15 21 22

However, it is worth noting that the sample consisting of adults with prelingual deafness comprised only 20 individuals, which is significantly fewer than the group with postlingual hearing loss, made up of 101 individuals. This is due to the fact that this population has more restricted indications for CI use compared with adults with postlingual deafness.

It was observed that 90 (89%) of participants with postlingual deafness had eCAP presence during the intraoperative period. The rate of satisfactory NRT results (defined as the presence of an electrical potential with clear morphology) in the present study was higher than that found by Guedes et al., 9 who reported eCAP presence in 72% of their sample, which, in turn, is lower than that described by Cafarelli-Dees et al., 23 who reported 96% eCAP presence during the postoperative period. In fact, the percentage of eCAP response presence in the postoperative period may be even higher, considering that some responses absent during the intraoperative phase may appear as present after continuous CI stimulation. 24

The higher rate of intraoperative eCAP response detection, compared with the findings reported by Guedes et al., 9 may be partly attributed to technological advances in CIs and to improved artifact cancellation methods available in more recent software. However, other factors may also have contributed to this difference, such as the clinical profile of the study population, the methodology employed, and the team's experience during the procedure.

Regarding the 11 (10%) participants with postlingual deafness exhibited absence of eCAP during the intraoperative period. The etiologies of hearing loss varied, including idiopathic hearing loss, viral infections, bacterial meningitis, traumatic brain injury, progressive deafness due to Usher syndrome, and genetic origins. Although the etiology of hearing impairment is described as one of the factors that can influence eCAP results as well as performance on speech recognition tests, the current study did not identify a specific pathology that could justify the absence of eCAP. 9 25 26 27

The average age of patients with absence of eCAP was 58 (range: 32–85) years. The duration of hearing deprivation reported by the patients averaged 17 (range: 1–70) years, 6 (54%) not effectively using the hearing aid in the preoperative period for the implanted ear, and only 1 participant underwent auditory rehabilitation prior to the CI.

One point that must be highlighted is that this sample included individuals with a maximum duration of hearing deprivation of 70 years, and only 6 individuals used the hearing aid effectively prior to surgery. This suggests that the absence of intraoperative eCAP may also be correlated with these factors, likely leading to a possible “deafferentation” of the auditory neural structures. 15 21 22 However, the data regarding the duration of hearing loss, as well as the effective use or non-use of the hearing aid, may not be entirely accurate, considering that it was collected based on patient-reported information.

The presence of eCAP in participants with postlingual hearing loss favored better performance in speech recognition tests compared with those who had no eCAP during the intraoperative period, both in closed-set speech tests ( *p* = 0.0046) and also in open-set presentation ( *p* = 0.0281). Although eCAP refers to peripheral neural structures, its presence indicates the preservation of ganglion cell structures that contribute to the conduction of the electrical stimulus, enhancing the processing of acoustic information. 9

However, the presence of neural response from the VIII cranial nerve during the surgical procedure was not sufficient to ensure good performance in speech recognition tests among individuals with prelingual hearing loss, as the vast majority performed only in closed-set conditions (75%). Likely due to cortical reorganization that, for many years, prioritized other sensory structures over hearing. 15 21 22 In this sample, all individuals with prelingual hearing loss showed improvement in auditory performance, with none achieving sentence recognition in open-set conditions prior to cochlear implantation. After implantation, only 4 patients (20%) demonstrated recognition in open-set presentations ranging from 20 to 50%.

The data indicate that, in addition to the duration of hearing loss, the age of onset also plays a crucial role in neural preservation. Studies have shown that early-onset hearing loss, particularly in the prelingual period, is associated with greater cortical reorganization and degeneration of central auditory pathways, even when peripheral stimulation is present. 15 21 22 Such reorganization may compromise the effectiveness of electrical stimulation via CIs, as the integrity of auditory pathways is essential for decoding the electrical signal. Conversely, individuals with late-onset hearing loss tend to exhibit better neural preservation, which may contribute to the superior performance observed in speech recognition tests among participants with postlingual deafness and present eCAP.

Despite the limited benefit of speech perception in prelingual individuals compared with postlingual ones, these individuals still report satisfaction and social independence with the use of CI. 28 29 However, the literature reports variability in speech recognition test results among individuals with prelingual hearing loss, with many achieving outcomes similar to those of individuals with postlingual hearing loss. 29

Therefore, understanding the behavior of pre-neural structures in individuals with pre- and postlingual hearing loss allows for a deeper understanding of the neural physiology of this population. It also enables the investigation of the relationship between the absence of eCAP during the intraoperative phase and the auditory behavior of these patients with cochlear implants, allowing for the adoption of more personalized programming and auditory rehabilitation measures for each individual.

Despite the relevant findings, the current study presents some limitations that should be considered when interpreting the results. The main limitation is related to the retrospective nature of the analysis, which relies on patient self-reported information, such as the duration of auditory deprivation and the effective use of hearing aids. Although all participants with prelingual deafness exhibited intraoperative neural responses (eCAP) and 89% of participants with postlingual deafness also showed eCAP during the procedure, it was not possible to determine whether this response was present in all electrodes or only in some, which may influence post-implant auditory performance. Furthermore, patients with absent intraoperative eCAP may eventually exhibit responses after continuous CI use, which could impact outcomes in speech perception tests.

Therefore, longitudinal studies are needed to evaluate the evolution of eCAP presence and its relationship with auditory outcomes. Future studies with a prospective design, larger sample sizes, and detailed analysis of eCAP distribution across electrodes may better clarify the relationship between peripheral neural integrity and auditory outcomes, particularly in populations with congenital deafness or prolonged sensory deprivation.

## Conclusion

It was found that among individuals with postlingual hearing loss, 89% had a neural response during the intraoperative CI surgery, and there was a correlation between the presence of eCAP and better performance in speech recognition tests. In individuals with prelingual hearing loss, 100% showed a neural response during the intraoperative; however, there was no correlation with performance in speech recognition tests.
